# Epigenetic therapies for neuroblastoma: immunogenicity awakens

**DOI:** 10.1002/1878-0261.13404

**Published:** 2023-03-08

**Authors:** Carlos Jiménez, Lucas Moreno, Miguel F. Segura

**Affiliations:** ^1^ Human Technopole Milan Italy; ^2^ Group of Childhood Cancer and Blood Disorders, Vall d'Hebron Research Institute (VHIR) Universitat Autònoma de Barcelona (UAB) Spain; ^3^ Paediatric Oncology and Haematology Department Vall d'Hebron University Hospital Barcelona Spain

**Keywords:** histone deacetylase inhibitors, immunotherapy, major histocompatibility complex class I, neuroblastoma

## Abstract

The development of immunotherapies for neuroblastoma remains challenging owing to the low immunogenicity of neuroblastoma cells, as reflected by the low expression of one of the main triggers of immune recognition, the major histocompatibility complex class I (MHC‐I). Cornel et al. showed that epigenetic modulation of neuroblastoma cells with a histone deacetylase inhibitor can boost the expression of major histocompatibility complex class I, among other immune receptors, priming their recognition by T‐ and natural killer cells. By leveraging the developmentally related aberrant epigenetic landscapes of neuroblastoma, these discoveries pave the way to overcome a major limitation in the field of neuroblastoma immunotherapy.

## Graphical abstract

Immunotherapies against the aggressive childhood tumour neuroblastoma remain challenged due to its low capacity of response to the immune system action. A recent work shows that that its immune responsiveness can be restored with entinostat, a molecule that promotes an epigenetic modulation that boosts the expression of immunogenic receptors, sensitising neuroblastoma cells to immunotherapy.

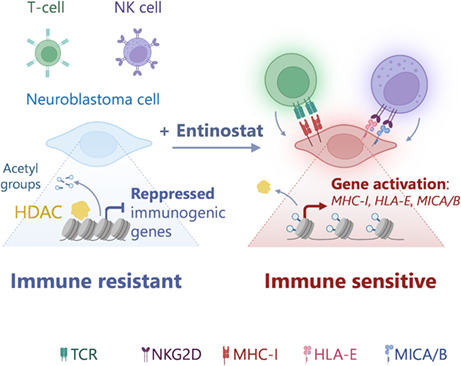

**Abbreviations**
CD137: cluster of differentiation 137
CTLA4: cytotoxic T lymphocyte‐associated antigen 4
HDACi: histone deacetylase inhibitors
HLA‐E: human leucocyte antigen E
IAPi: inhibitors of apoptosis proteins
IFN‐α: interferon alpha
IFN‐γ: interferon gamma
MHC‐I: histocompatibility complex class I
MIBG: metaiodobenzylguanidine
MICA/B: MHC class I chain‐related protein A and B
N4BP1: NEDD4 binding protein 1
NFκB: nuclear factor kappa light chain enhancer of activated B cells
NK: natural killer
PD‐1: programmed cell death protein 1
PRAME: preferentially expressed antigen in melanoma
RNAseq: RNA sequencing
TNFα: tumour necrosis factor alpha

High‐risk neuroblastoma remains a clinical challenge worldwide, as this group of patients, representing close to half of all cases of neuroblastoma, still presents 5‐year survival rates below 50% [1]. Although the survival of patients with these peripheral nervous system neoplasms has partially benefited from certain immunotherapies (i.e. anti‐GD2 therapy), some immunotherapies with promising results in adults such as anti‐PD1 or anti‐CTLA4 do not have the expected results in children [2]. Thus, these above‐mentioned immunotherapies are limited by the low immunogenicity of neuroblastoma cells [3]. A paradigmatic example reflecting this feature is the absence or low expression of MHC‐I in neuroblastoma cells [4].

MHC‐I is expressed in all nucleated cells of post‐embryonic tissues, and its expression is a key requirement for the recognition of neoplasms and surveillance by the immune system. Loss of expression of this antigen‐presenting membrane receptor is a recurrent immune‐evading survival strategy of cancer cells, through mutations or gene silencing [5, 6]. However, tumours of embryonal origin such as neuroblastoma originate from early embryonal cell populations not expressing MHC‐I. In this case, the lack of expression is not due to a clonal selection process leading to its loss, but to the specific gene expression programming of the cell of origin, most usually through epigenetic mechanisms [7].

Neuroblastoma, as a paediatric cancer, harbours low mutational rates. However, increasing evidence emphasises the importance of the aberrant epigenomes of neuroblastoma cells due to their oncogenic properties. Neuroblastoma cells carry “frozen‐in‐time” epigenetic landscapes that reflect their embryonal origins and resemble those of the neural crest progenitors from which they were originated [8]. Therefore, understanding neuroblastoma epigenetics allows hacking these expression programs and reverting relevant oncogenic functions. In the case of immune evasion, the developmental inherited and reversible epigenetic silencing of MHC‐I, contrary to genetic loss in non‐embryonal tumours, opens a promising opportunity for re‐expressing MHC‐I to induce neuroblastoma responsiveness to the immune system.

To revert one of the main mechanisms of immune evasion of neuroblastoma cells, Cornel et al. [9] sought to screen for compounds that would restore the expression of MHC‐I. The authors used two drug repurposing screening libraries, testing approximately 3900 compounds in a range of nanomolar to low micromolar concentrations. To dissect the mechanism of the drug tested, all these compounds were probed in a neuroblastoma cell line containing an NFκB gene reporter system, as the authors and others had shown before that this pathway mediates the upregulation of the MHC‐I gene induced by cytokines [10, 11]. Among all drugs tested, two families of compounds stood out: histone deacetylase inhibitors (HDACi) and inhibitors of Inhibitors of apoptosis proteins (IAPi), both of which enhanced MHC‐I expression in an NFκB‐independent and NFκB‐dependent manner, respectively. The functional consequences of this drug‐mediated induction of MHC‐I were tested in a co‐culture system consisting of neuroblastoma cell lines and T‐cells directed against the neuroblastoma‐specific Preferentially Expressed Antigen in Melanoma (PRAME) antigen. The IAPi AZD‐5582 was able to induce the expression of MHC‐I and T‐cell‐mediated toxicity in a few neuroblastoma cell lines, owing to the strong expression of negative modulators of the NFκB pathway, a common feature of neuroblastoma tumours [11]. The authors elegantly showed that silencing of N4BP1, a well‐known NFκB inhibitor, allowed AZD‐5582‐mediated induction of MHC‐I.

Because the effects of HDACi on MHC‐I were not mediated by NFκB, the authors further focused on the HDACi entinostat, a drug that is currently being tested in children with relapsed or refractory malignancies in combination with nivolumab (NCT03838042). Contrary to what was seen with IAPi, entinostat was able to induce MHC‐I expression in a large variety of standard neuroblastoma cell lines as well as in patient‐derived organoids, across the diverse spectrum of genomic aberrations associated with neuroblastoma. Increase of MHC‐I was also observed with other HDACi and was further enhanced by combinatorial treatment using cytokines known to regulate MHC‐I.

The functional consequences of entinostat treatment were analysed measuring the activation (via CD137 expression as well as TNFα and IFN‐γ secretion) and cytotoxic capacity of T‐cells. Pre‐treatment with entinostat alone was sufficient to increase the expression of CD137, and this effect was further enhanced in the presence of other T‐cell activation cytokines such as IFN‐α and IFN‐γ. The consequences of T‐cell activation were analysed in a cytotoxic assay, which showed that entinostat pre‐treatment resulted in a similar increase in the cytotoxic capacity of T‐cells as pre‐treatment with other MHC‐I‐enhancing cytokines. The fact that experiments were performed in the presence of HDACi suggests that entinostat did not affect the viability and cytotoxic capacity of T‐cells.

Because the effects of HDAC inhibition may alter many cellular processes, the authors further investigated whether the immunogenic effects of HDACi could be attributed to other factors. A whole transcriptomic analysis (by RNAseq) was performed to analyse differentially expressed genes upon entinostat treatment. Interestingly, the authors found a significant modulation of genes not only involved in MHC‐I antigen presentation but also genes encoding natural killer (NK) signalling molecules. A fraction of these genes was also demonstrated to be upregulated and present on the surface of the plasma membrane. Co‐culture cytotoxicity experiments showed that entinostat pre‐treatment of neuroblastoma cells increased the cytotoxic capacity of healthy‐donor NK cells. In summary, these findings indicate that the immunogenic effects of entinostat treatment are beyond the upregulation of MHC‐I.

Intriguingly, Cornel et al. also observed that entinostat promoted a switch towards the mesenchymal lineage of neuroblastoma cells at the transcriptional level, accompanying the increase in genes related to immune activation. Indeed, recent studies have revealed that the immunogenic status of neuroblastoma is related to the adrenergic (less immunogenic) and mesenchymal (more immunogenic) cell lineages widely studied recently in these tumours [12]. The biological reason for the higher immunogenicity of the more invasive and therapy‐resistant mesenchymal phenotype remains elusive. However, this discovery revealed a key vulnerability of this subpopulation of aggressive neuroblastoma cells that could be exploited with entinostat treatment, as proposed by the authors.

In summary, although T‐cell dysfunction may be caused by multiple factors, the present study demonstrated a clear effect of HDACi on the immunogenic capacity of neuroblastoma cells by releasing the developmental epigenetic brake that blocks the expression of immune‐related genes (Fig. 1). Validation in animal models showing the enhanced efficacy of immunotherapies in combination with HDACi will strength the conclusions of this study.

**Fig. 1:**
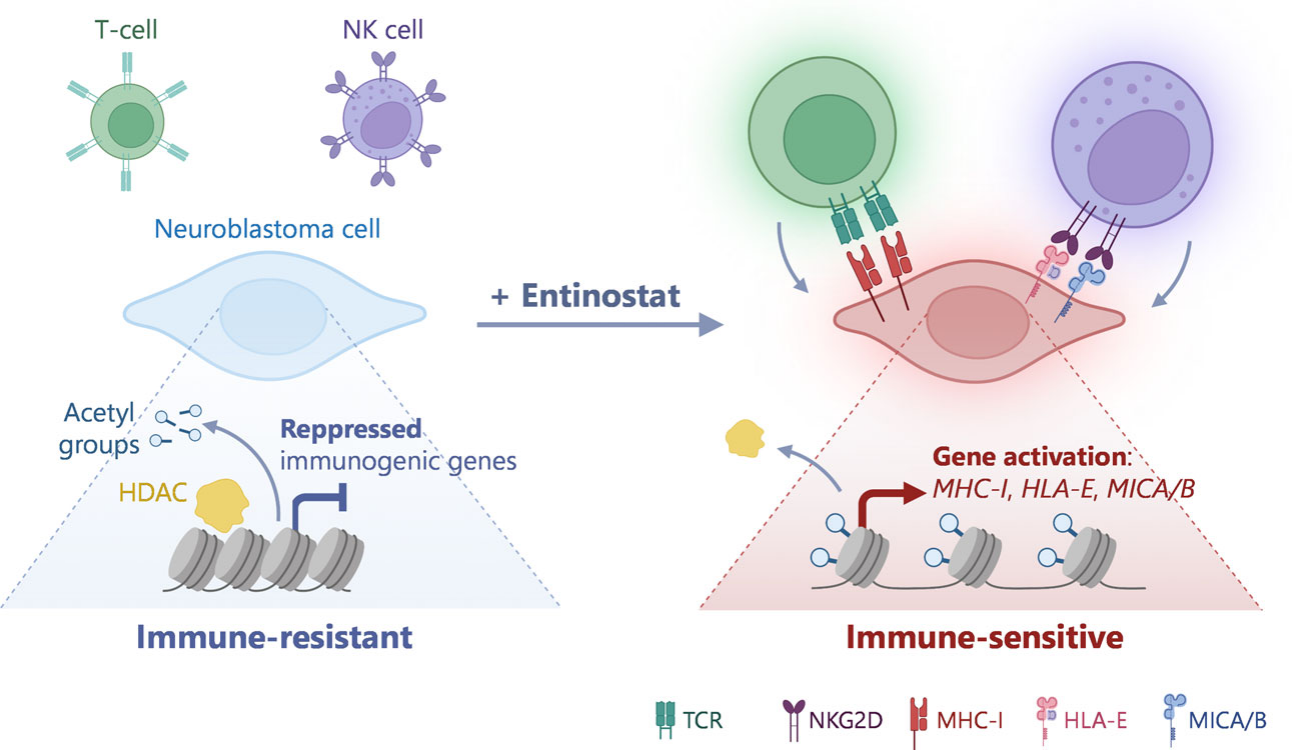
Immunogenic activation of neuroblastoma cells through epigenetic therapy. Cornel et al. showed in their recent work that the expression of multiple immune system receptors, epigenetically silenced in neuroblastoma cells as a result of its developmental origins, can be restored with the histone deacetylase (HDAC) inhibitor entinostat. This epigenetic modulation boosts the expression of major histocompatibility complex class I (MHC‐I), key for T‐cell recognition, and human leukocyte antigen E (HLA‐E) and MHC class I chain‐related proteins A and B (MICA/B), receptors of natural killer (NK) cells, thereby triggering a cytotoxic immune response against neuroblastoma cells.

Although similar results have been observed in adult tumours, it is important to highlight that this therapeutic approach could benefit other embryonal tumours, thereby supporting the rationale of combining epigenetic and immune‐based therapies in ongoing and future paediatric cancer clinical trials. These trials will be fundamental to demonstrate the feasibility of activating the pro‐immunogenic gene expression program at clinically relevant doses with manageable toxicities and potentiate the use of new combinations of immunotherapies with standard approaches such as metaiodobenzylguanidine (MIBG) therapy or chemotherapy.

## Conflict of interest

The authors declare no conflict of interest.
